# Electroacupuncture relieves portal hypertension by improving vascular angiogenesis and linking gut microbiota in bile duct ligation rats

**DOI:** 10.3389/fmicb.2023.1207137

**Published:** 2023-07-11

**Authors:** Po-Yu Huang, Hsuan-Miao Liu, Yan-Ru Ko, Zi-Yu Chang, Tzung-Yan Lee

**Affiliations:** ^1^Graduate Institute of Clinical Medical Sciences, College of Medicine, Chang Gung University, Taoyuan, Taiwan; ^2^Department of Chinese Medicine, Linsen Chinese Medicine and Kunming Branch, Taipei City Hospital, Taipei, Taiwan; ^3^Graduate Institute of Traditional Chinese Medicine, School of Chinese Medicine, College of Medicine, Chang Gung University, Taoyuan, Taiwan; ^4^Department of Traditional Chinese Medicine, Chang Gung Memorial Hospital, Keelung, Taiwan

**Keywords:** electroacupuncture, portal hypertension, gut microbiota, vasculopathy, angiogenesis, ST36

## Abstract

The pathological increase in the intrahepatic resistance and decrease peripheral vascular tone in the development of portal hypertension (PHT). PHT has been linked to lower microbial diversity and weakened intestinal barrier, and interplay alters inflammatory signaling cascades. Electroacupuncture (EA) may ameliorate the inflammatory response and limit arterial vasodilatation and portal pressure. This study addresses the possible mechanisms underlying putative hemodynamics effects of EA in PHT rats. PHT was induced by bile duct ligation (BDL) over 7 days in rats. BDL rats were treated with low-frequency EA (2 Hz) at acupoint, ST36, 10 min once daily for 7 consecutive days. EA significantly reduced portal pressure and enhanced maximum contractile responses in the aorta, and blunts the angiogenesis cascade in PHT rats. EA decreased the aortic angiogenesis signaling cascade, reflected by downregulated of ICAM1, VCAM1, VEGFR1, and TGFβR2 levels. In addition, EA preserved claudin-1, occludin, and ZO-1 levels in BDL-induced PHT model. Furthermore, EA demonstrates to have a positive effect on the gut *Bacteroidetes*/*Firmicutes* ratio and to reduce pro-inflammatory cytokines and endotoxins. These results summarize the potential role of EA in the gut microbiota could potentially lead to attenuate intestine injury which could further contribute to vascular reactivity in PHT rats.

## 1. Introduction

Portal hypertension (PHT) is a condition resulting from an increase in resistance of the portal venous system due to increase in intrahepatic resistance and/or obstruction of the portal venous system (Abraldes et al., 2019). This resistance is caused by increased pressure in the portal vein, which is usually due to increased resistance of the intrahepatic blood vessels. This increased resistance of the intrahepatic vessels is caused by the accumulation of portal-systemic collaterals (PSCs) and hepatic fibrosis, both of which are caused by chronic liver disease (Bosch et al., 2008). These changes in the intrahepatic vessels can lead to further caused by a blockage in the blood vessels that lead from the liver to the heart. All of these mechanisms can lead to the development of PHT and its associated in vascular hyporeactivity (Dadhich et al., 2014). This is a reduction in the responsiveness of the blood vessels to normal stimuli, resulting in increased resistance to blood flow. This increased resistance results in an increase in the pressure within the portal vein, which in turn leads to the development of PHT. The exact cause of vascular hyporeactivity is not fully understood but is thought to be related to the presence of pathological structural changes in the walls of the vessels. These changes are associated with increased inflammation and angiogenesis of the vessel wall (Bosch et al., 2008; Dadhich et al., 2014). These results in an impairment of the ability of the vessel to dilate, which in turn increases the resistance to blood flow (Dadhich et al., 2014).

The microbiota has been increasingly recognized for its role in the development and progression of PHT (Gedgaudas et al., 2022). Numerous studies have identified alterations of the gut microbiota that may contribute to vascular hyporeactivity, a signature of PHT (Baffy, 2019; Gedgaudas et al., 2022). These changes include a shift in the ratio of *Firmicutes* to *Bacteroidetes*, an increase in the abundance of *Enterobacteriaceae*, and a decrease in the abundance of obligate anaerobes (Baffy, 2019). Furthermore, the composition of the microbiota has been associated with portal pressure (Gedgaudas et al., 2022) and hepatic fibrosis (Nie et al., 2021), two major drivers of PHT. It is hypothesized that the microbiota affects PHT and vascular hyporeactivity through the release of metabolites that modulate tissue inflammation and immune responses. Further research is needed to better understand the relationship between the microbiota and PHT, and to identify potential therapeutic strategies for reversing vascular hyporeactivity.

Acupuncture is a traditional Chinese practice that involves inserting thin metal needles into the body at specific points to stimulate the flow of energy, or qi. It is thought to have been used for over 3,000 years in Asia, and is believed to provide relief from a wide variety of ailments and medical conditions (Nie et al., 2021). Acupuncture is now used widely in the United States and other countries, and is often used to treat a variety of medical issues ranging from digestive problems, to chronic pain, to stress (Greenlee et al., 2017). Studies have also found that acupuncture can provide relief from chronic pain, fibromyalgia, headaches, and even migraines (Kelly, 2009; Fogleman and McKenna, 2022). Overall, acupuncture is a safe, non-invasive practice that has been used for thousands of years to address a variety of medical issues. Although it is not a cure-all, it can provide relief to those who are suffering from a variety of diseases conditions. Electroacupuncture (EA) is a form of acupuncture that uses electrical stimulation to apply acupuncture needles to specific points on the body. In the case of PHT, EA has been used to reduce portal pressure and improve vascular function (Chen et al., 2022). In the case of non-alcoholic fatty liver disease (NAFLD), EA has been used to reduce liver fat, improve liver enzymes, and reduce inflammation (Wen and Lee, 2014, 2015). These animal studies have shown that EA is effective in reducing lipogenesis signaling of liver and adipose tissue, improving liver enzymes and reducing inflammation in adipose tissue with NAFLD. In the case of hypertension, EA has been used to reduce blood pressure in patients with hypertension (Fan et al., 2020). Finally, EA may stimulate the release of neurotransmitters, including vasoactive intestinal peptide (VIP), which is known to regulate the activity of the autonomic nervous system. In our previous experiment, EA was used to stimulate the release of VIP, which then activates the vasoactive intestinal peptide receptor 2 (VPAC2). This receptor then triggers a cascade of signals that increase inflammation-regulating molecules and cytokines, which further regulate intestinal mucosal immunity, which helps to reduce inflammation and maintain intestinal health (Liu et al., 2022). This process demonstrates how EA stimulation can have an anti-inflammatory effect and can be used to promote intestinal health.

The treatment for PHT involves a multi-faceted approach that should address both vascular activity and microbiome. The major goal is to reduce the portal pressure. Therefore, EA may be performed to reduce portal pressure, but the effect of acupuncture on intestinal microbes is unknown. We set the primary goal of this research is to analyze the impact of EA treatment on the hemodynamics of rats with BDL-induced PHT. The second goal of this study is to investigate the effects of EA on endothelium-dependent relaxation and its ability to modulate angiogenic factors, and its role in preventing arterial hyporeactivity to vasoconstrictors. The microbiome of the gut is also an important factor in the treatment of PHT. An imbalance in the gut microbiome can lead to an increase in proinflammatory cytokines and growth factors, which can contribute to the development of PHT. Therefore, EA may be performed, as well as other interventions, can be beneficial in restoring the balance of the microbiome and improving PHT symptoms. Finally, the role of EA in PHT-induced imbalance of the microbiome is also examined.

## 2. Materials and methods

### 2.1. Animals experiment design

Male Sprague–Dawley rats weighing 200–250 g, were obtained from National Laboratory Animal Center (Taipei City, Taiwan). Hepatic common BDL-induced PHT was produced as previously described (Chen et al., 2022). In brief, under isoflurane (2% in 1:1 mixture of oxygen and air) for about 20–30 min, the common bile duct was ligated with 3–0 silk and sectioned between the ligatures. The midline abdominal incision was closed with catgut. Sham operated rats had their bile duct exposed but not ligated or sectioned. All rats were caged at 25°C under a 12:12 h lightdark cycle and were allowed free access to food and water. Animal studies were approved by the Animal Experiment Committee of the Chang Gung University (IACUC approval no. CGU15-088) and were conducted humanely. A total of 15 rats were divided into three groups (*n* = 5 per group): (1) sham; (2) BDL; (3) BDL + EA36. Before needle insertion, the rats were lightly anesthetized with isoflurane (2% in 1:1 mixture of oxygen and air) for about 20–30 min. Sham-operated and BDL rats were treated with low-frequency EA (2 Hz) at acupoint, ST36, 10 min once daily for 7 consecutive days. Stainless steel acupuncture needles (0.5 in., 32 gage) were inserted to a depth of 0.3 cm into the muscle layer at ST36 and stimulated using an EA apparatus (Digitimer DS3, Letchworth Garden City, United Kingdom) at 2 Hz frequency with 0.1-s (Liu et al., 2018). The intensity varied from 0.5 to 1.0 mA during stimulation and was adjusted to produce local muscle contraction.

### 2.2. Hemodynamic studies

After administering isoflurane to all the rats, hemodynamic tests were performed out on rats. A polygraph (RS3400, Gould, Valley View, OH, United States) was used to monitor changes in pressures and heart rate using strain-gage transducers (P23XL, Viggo-Spectramed, Oxnard, CA, United States). The right femoral artery and ileocolic vein were directly cannulated to measure the mean arterial pressure (MAP) and portal venous pressure (PVP), respectively. A pulse Doppler probe (T-206, Transonic System Inc., Ithaca, NY, United States) was wrapped around the isolated segment of the superior mesenteric artery (SMA) to monitor blood flow through it. Following isoflurane anesthesia, hemodynamic data were recorded in all experimental groups. Blood samples (from the femoral artery) were taken after the hemodynamic assessments and immediately centrifuged at 4°C. Until assays were conducted, the resulting serum was stored at −20°C.

### 2.3. *In vitro* vascular contractility study

Animals were euthanized using isoflurane and exsanguinated by cardiac puncture, then the thoracic aorta was prepared as previously described (Chen et al., 2022). To determine isometric tension, aortic and SMA rings were equilibrated in a 10 mL tissue chamber maintained at 37°C and bubbled with a gas mixture of 95% oxygen and 5% carbon dioxide. The consistency of the responses elicited by three successive tests with 60 mM KCl revealed the tissue’s preparedness. Then, cumulative concentration-response curves for phenylephrine (10^−9^–10^−5^ M) were obtained. After 45 min of recuperation and rinsing, the tissue was used to create the previously described contractile response curves to phorbol-12,13-dibutyrate (PDBu; 3 × 10^−6^ M). The tissue was subjected to a challenge of 60 mM KC1 at the end of the experiment (Huang et al., 1996).

### 2.4. Histopathology, immunohistochemistry, and immunofluorescence staining

Thoracic aorta was fixed in 10% formalin, embedded in paraffin, and then cut into 5-μm-thick slices and stained with hematoxylin–eosin (H&E). A pathologist subsequently used light microscopy to analyze the sections. Xylene and subsequent ethanol baths were used to dewax and rehydrate paraffin slices. After thorough washings in phosphate-buffered saline (PBS), tissue sections were incubated with certain primary antibodies for 2 h at room temperature before being thoroughly rinsed three times in PBS. For immunohistochemistry (IHC) and immunofluorescence (IF) studies, the following antibodies were used: intercellular adhesion molecule1 (ICAM1; GTX22213, GeneTex, CA, United States), vascular cell adhesion protein 1 (VCAM1; ab134047, Abcam, Cambridge, United Kingdom), vascular endothelial growth factor receptor 1 (VEGFR1; ab32152, Abcam, Cambridge, United Kingdom), transforming growth factor beta receptor 2 (TGFβR2; ab32152, Abcam, Cambridge, United Kingdom), Claudin1 (ab307692, Abcam, Cambridge, United Kingdom), Occludin (ab216327, Abcam, Cambridge, United Kingdom), zonula occludens 1 (ZO1; ab190085, Abcam, Cambridge, United Kingdom), and farnesoid X receptor (FXR; ab51970, Abcam, Cambridge, United Kingdom).

Samples were incubated with peroxidase- or fluorescent compound–conjugated secondary antibodies for 1 h at 37°C followed by extensive washes in PBS. Images were visualized using Olympus IX71 Inverted Fluorescence Microscope (Olympus, Japan). Representative images were analyzed using Olympus cellSens software (Olympus, Japan).

### 2.5. Biochemical parameter analysis

Rat uncoated ELISA kit (Thermo Fisher Scientific, MA, United States) was used to perform tests on serum levels of TNF alpha, MCP-1, and IL-1β as previously described (Wang et al., 2021).

### 2.6. Quantitative real-time reverse transcription PCR assay

Total RNA was extracted using 1 mL of TRIzol (Thermo Fisher Scientific, MA, United States) from 0.2 g aorta tissues. The cDNA was reverse transcribed using a High-Capacity cDNA Reverse Transcription Kit (Thermo Fisher Scientific, MA, United States) from RNA according to the manufacturer’s instructions. The cDNA samples were amplified by qRT-PCR using SYBR Green I (Roche, Switzerland). In qRT-PCR, SYBR Green I was used to amplify the cDNA samples (Roche, Switzerland). For qPCR, the thermocycling conditions were applied as previously described (Wang et al., 2021). As a housekeeping gene, GAPDH was tested to verify the stability in this experimental model. Therefore, in this study, gene expression levels were normalized to those of GAPDH. The primer sequences used in this study are *Tnfα*: forward, 5′ tcagttccatggcccagac 3′, reverse, 5′ gttgtctttgagatccatgccatt 3′ (NM_012675.3); *Mcp1*: forward, 5′ tagcatccacgtgctgtctc 3′, reverse, 5′ cagccgactcattgggatca 3′ (NM_031530.1); *Il-1β*: forward, 5′ aacctgctggtgtgtgacgttc 3′, reverse, 5′ cagcacgaggcttttttgttgt 3′ (NW_047658); and *Gapdh*: forward, 5′ ggcacagtcaaggtgagaatg 3′, reverse, 5′ atggtggtgaagacgccagtc 3′ (NM_017008.3).

### 2.7. Western blot analysis

Tissues were homogenized in T-PER™ Tissue Protein Extraction Reagent (0.30 mg tissue/200 μL; Thermo Fisher Scientific, MA, United States) or NE-PER nuclear and Cytoplasmic Extraction Reagents (Thermo Fisher Scientific, MA, United States) containing proteinase inhibitors (1 μL/mL; Sigma-Aldrich; Merck KGaA, Rahway, NJ, United States). Protein (60–80 g/lane) was run on a 10% SDS-PAGE gel and then transferred to a PVDF membrane after the soluble proteins were measured using the Bio-Rad Rapid Coomassie kit (Bio-Rad Laboratories, Hercules, California, United States; GE Healthcare, Chicago, Illinois, United States). Pierce™ Fast Blocking Buffer (Thermo Fisher Scientific, MA, United States) was used for blocking at room temperature for 1 h. The PVDF membranes were incubated with primary antibody in Pierce™ Fast Blocking Buffer at 4°C with gentle shaking overnight, and then the secondary antibody was incubated at room temperature for 1 h. The protein expression was detected using an enhanced chemiluminescence kit and quantified using ImageJ 1.53 software (National Institutes of Health, Maryland, United States). Primary antibodies involved in this study includes: ICAM1 (GTX22213, GeneTex, CA, United States), VCAM1 (ab134047, Abcam, Cambridge, United Kingdom), VEGFR1 (ab32152, Abcam, Cambridge, United Kingdom), TGFβR2 (ab32152, Abcam, Cambridge, United Kingdom), Claudin1 (ab307692, Abcam, Cambridge, United Kingdom), Occludin (ab216327, Abcam, Cambridge, United Kingdom), ZO1 (ab190085, Abcam, Cambridge, United Kingdom), FXR (ab51970, Abcam, Cambridge, United Kingdom), and Histone (ab1791, Abcam, Cambridge, United Kingdom).

### 2.8. Gut microbiota sequencing and microbial analysis

E. Z.N.A. ®Stool DNA Kit (D4015, Omega, Inc., United States) was used to extract genomic DNA from fecal samples in accordance with the manufacturer’s recommendations. A 341F/805R primer pair was used to conduct amplicons of the V3-V4 region of the 16S rDNA gene. A NovaSeq PE250 platform was used for the sequencing. Quantitative Insights Into Microbial Ecology2 (QIIME2) and R programs were mostly used for the analyses of sequence data (v3.5.2). Feature table and feature sequence were retrieved by DADA2 for dereplication. Only the sequences between 250 and 450 bases long with an end-trimming quality better than 25 and analyses performed in windows of 50 bases were considered for taxonomy categorization after chimeric sequences were ruled out. Using the RDP database (Ribosomal Database Project) for the 16S rRNA gene with a bootstrap cut-off of 80%, these high quality partial sequences of the 16S rRNA gene were classified in operational taxonomic units (OTUs). Only the OTUs representing over 0.1% of the total sequences of each sample were considered in the subsequent statistical analyses.

### 2.9. Statistics

The Statistical Package for the Social Sciences (SPSS) version 21.0 (SPSS Inc., Chicago, IL, United States) was applied to conduct the statistical analysis. The Kruskal-Wallis test was employed to examine all data, which were reported as mean ± standard error of mean (SEM). The difference at each time point was calculated using the Mann–Whitney U-test at a significance level of *p* < 0.05.

## 3. Results

### 3.1. Hemodynamic parameters after EA36 treatment in BDL rats

**Figure 1:**
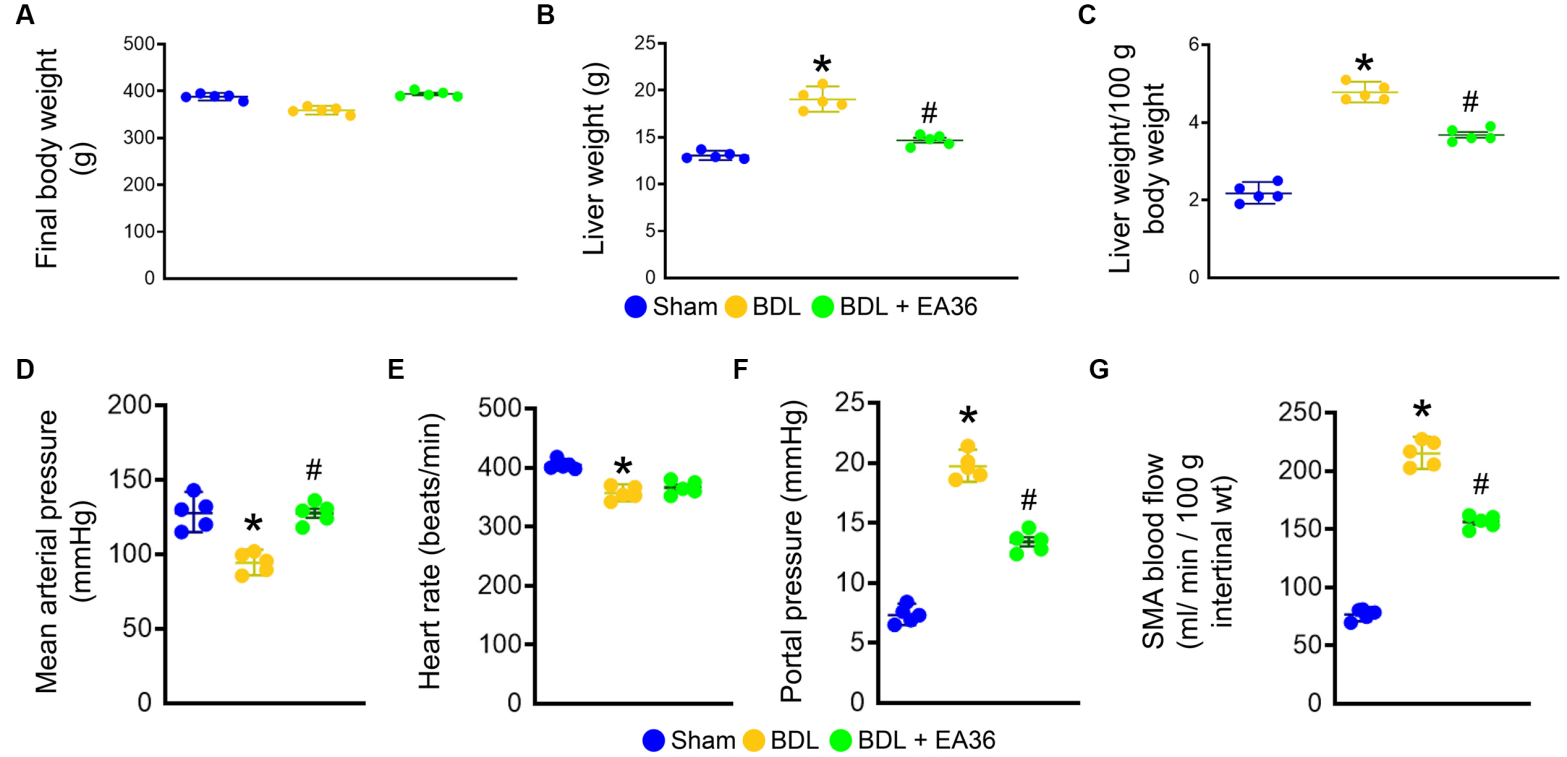
Effects of EA36 on hemodynamic values in a rat model of portal hypertension induced by bile duct ligation. **(A)** Final body weight (g). **(B)** Liver weight (g). **(C)** Liver weight/100 g body weight. **(D)** Mean arterial pressure (mmHg). **(E)** Heart rate (beats/min). **(F)** Portal pressure (mmHg). **(G)** SMA blood flow (mL/min/100 g intertinal wt). The results represent mean ± SEM. ^*^p < 0.05, sham compared with BDL; ^#^*p* < 0.05, BDL compared with BDL + EA36.

We first evaluated the hemodynamic parameters in sham rats, BDL rats and BDL rats treated with EA36 (Figure 1). The final bodyweight was not significantly different between the groups (Figure 1A). In comparison to sham rats, liver weight, liver weight/100 g body weight, portal pressure and SMA blood flow were considerably increased in BDL rats (Figures 1B,C,F,G). Both mean arterial pressure and heart rate were lower in BDL rats, whereas EA36 only increased mean arterial pressure while having no effect on heart rate (Figures 1D,E). In contrast, the liver weight, liver weight/100 g body weight, portal pressure, and SMA blood flow values in BDL rats were also reversed by EA36.

### 3.2. Effect of EA36 on angiogenesis and inflammation levels in rats with PHT

**Figure 2:**
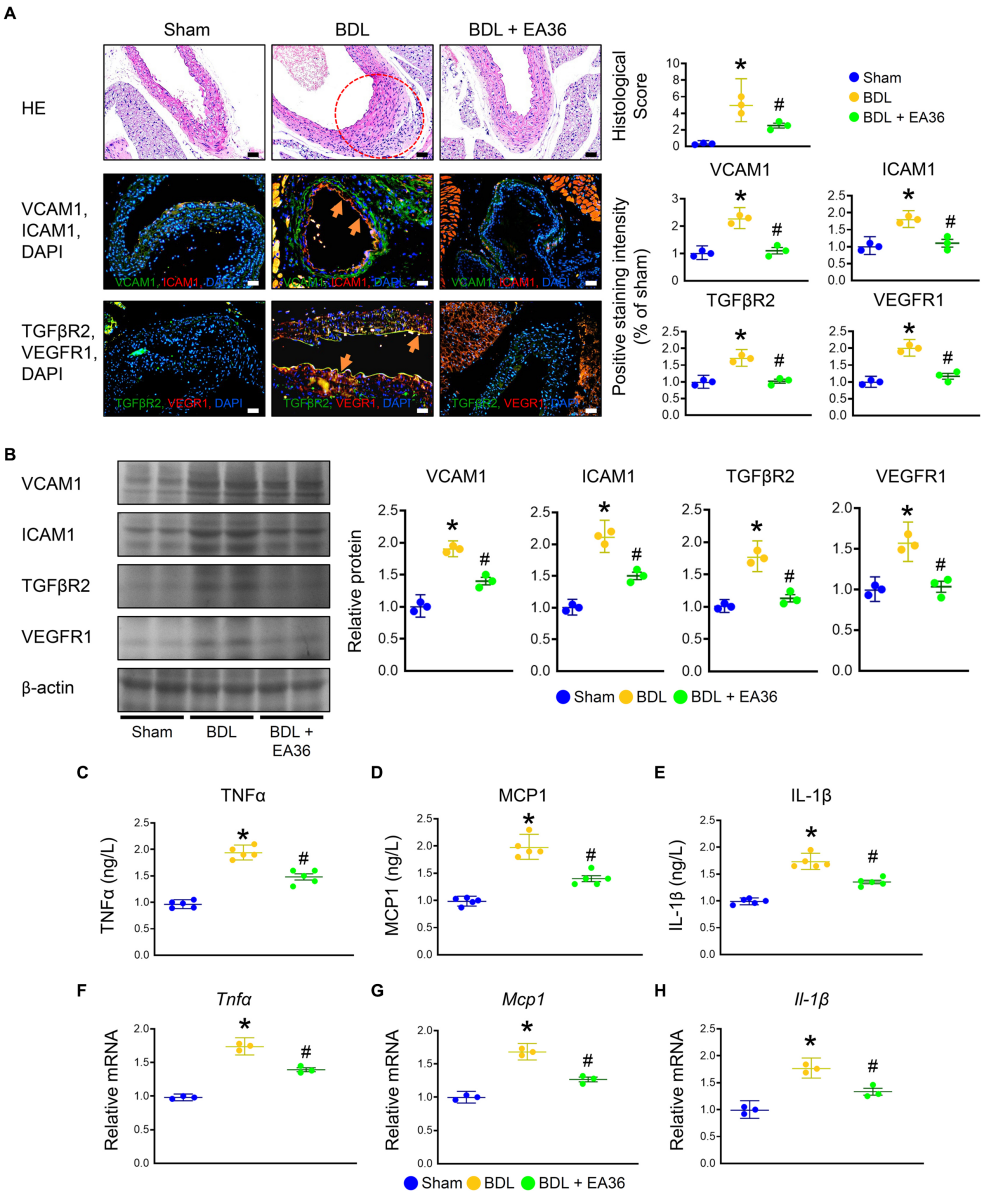
Effects of EA36 on angiogenesis and inflammation levels in a rat model of portal hypertension induced by bile duct ligation. **(A)** Effects of electroacupuncture (EA36) treatment on angiogenesis signaling in BDL rats relative to untreated sham and BDL rats. The red circles denote aortic damage. Aortas were sectioned and double immunofluorescence was performed with ICAM1 (green) and VCAM1 (red), as well as, TGFβR2 (green) and VEGFR1 (red). Angiogenesis-positive cells were observed adjacent to the vascular endothelium in the aorta of BDL rats. Use DAPI staining solution to stain DNA in fluorescent tissue imaging. Orange arrows highlight positive staining. Scale bar: 50 μm. **(B)** Quantification of VCAM1, ICAM1, TGFβ2, and VEGFR1 protein levels by western blot of aorta. Right graphs indicate quantification relative to β-actin. **(C–E)** Concentration of TNFα, MCP1, and Il-1β in serum. **(F–H)** Quantification of *Tnfα*, *Ifnγ*, Mcp1, and *Il-1β* by qRT-PCR in aorta. qRT-PCR indicates quantification relative to Gapdh. The results represent mean ± SEM. ^*^p < 0.05, sham compared with BDL; ^#^*p* < 0.05, BDL compared with BDL + EA36.

There was a marked increased in the abundance of ICAM1, VCAM1, TGFβR2, and VEGFR1 in PHT, when comparing BDL versus sham groups. Based on immunofluorescence (IF) and western blot (WB) analyses, treatment with EA36 inhibited angiogenesis, correlated with the diminution of ICAM1, VCAM1, TGFβR2, and VEGFR1 in the aortas of BDL rats (Figures 2A,B). In addition, BDL rats had higher TNFα, MCP1, and IL-1β levels in serum (Figures 2C–E), and Tnfα, Mcp1, and IL-1β mRNA expression in aortas (Figures 2F–H); after EA36 treatment, the proinflammatory cytokines were significantly reduced compared with BDL rats.

### 3.3. EA36 enhances contractile responses in aorta and SMA rings

**Figure 3:**
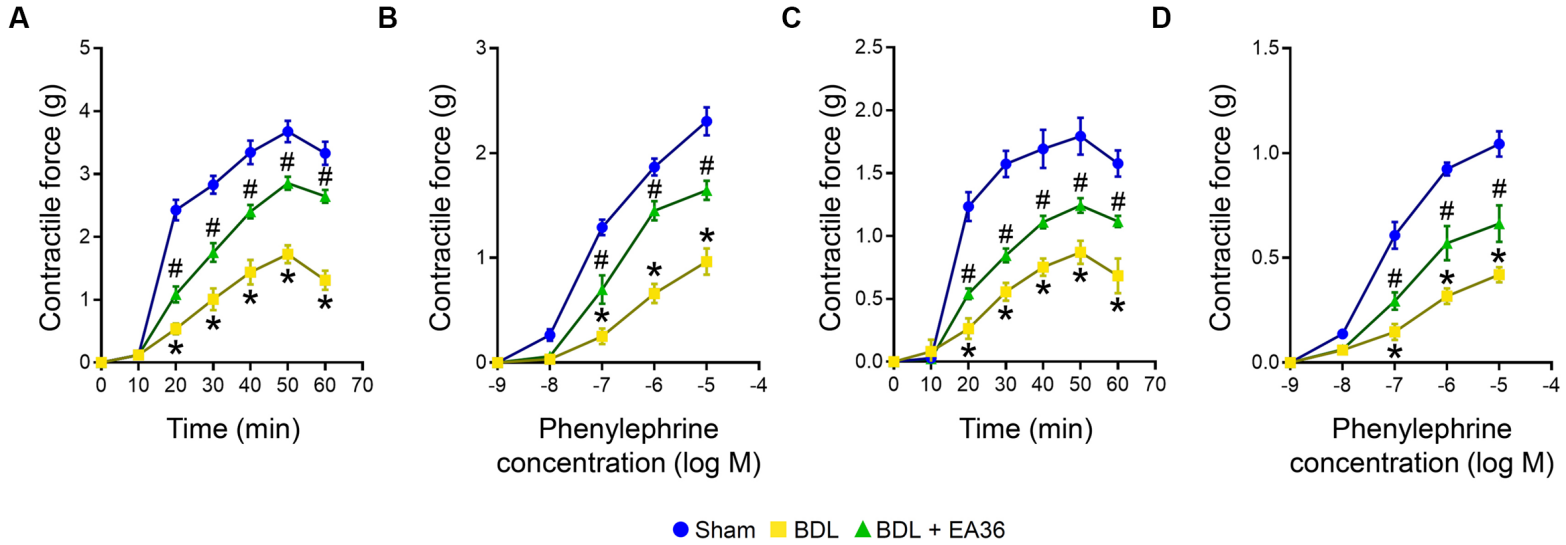
Effects of EA36 on contractile responses of aortic and superior mesenteric arterial in BDL rats. **(A,B)** Contractile responses of aortic and **(C,D)** superior mesenteric arterial rings to phorbol-12,13-dibutyrate (PDBu) and concentration-response curves of phenylephrine in the aorta and superior mesenteric artery. The results represent mean ± SEM. ^*^p < 0.05, sham compared with BDL; ^#^*p* < 0.05, BDL compared with BDL + EA36.

Compared to the BDL groups, the contractile responses were considerably higher in the EA36-treated groups (Figure 3). The maximal contraction induced by PDBu (3 × 10^−6^ M) was significantly lower in BDL rat aortas (Figure 3A) and SMA rings (Figure 3C). In comparison to the sham group, the maximum contraction brought on by phenylephrine in the aorta of BDL rats was less pronounced. In the aorta and SMA rings during EA36 treatment, the maximum contraction was similarly, dramatically higher than in the BDL group (Figures 3B,D).

### 3.4. EA36 ameliorated intestinal barrier damage in BDL rats

**Figure 4:**
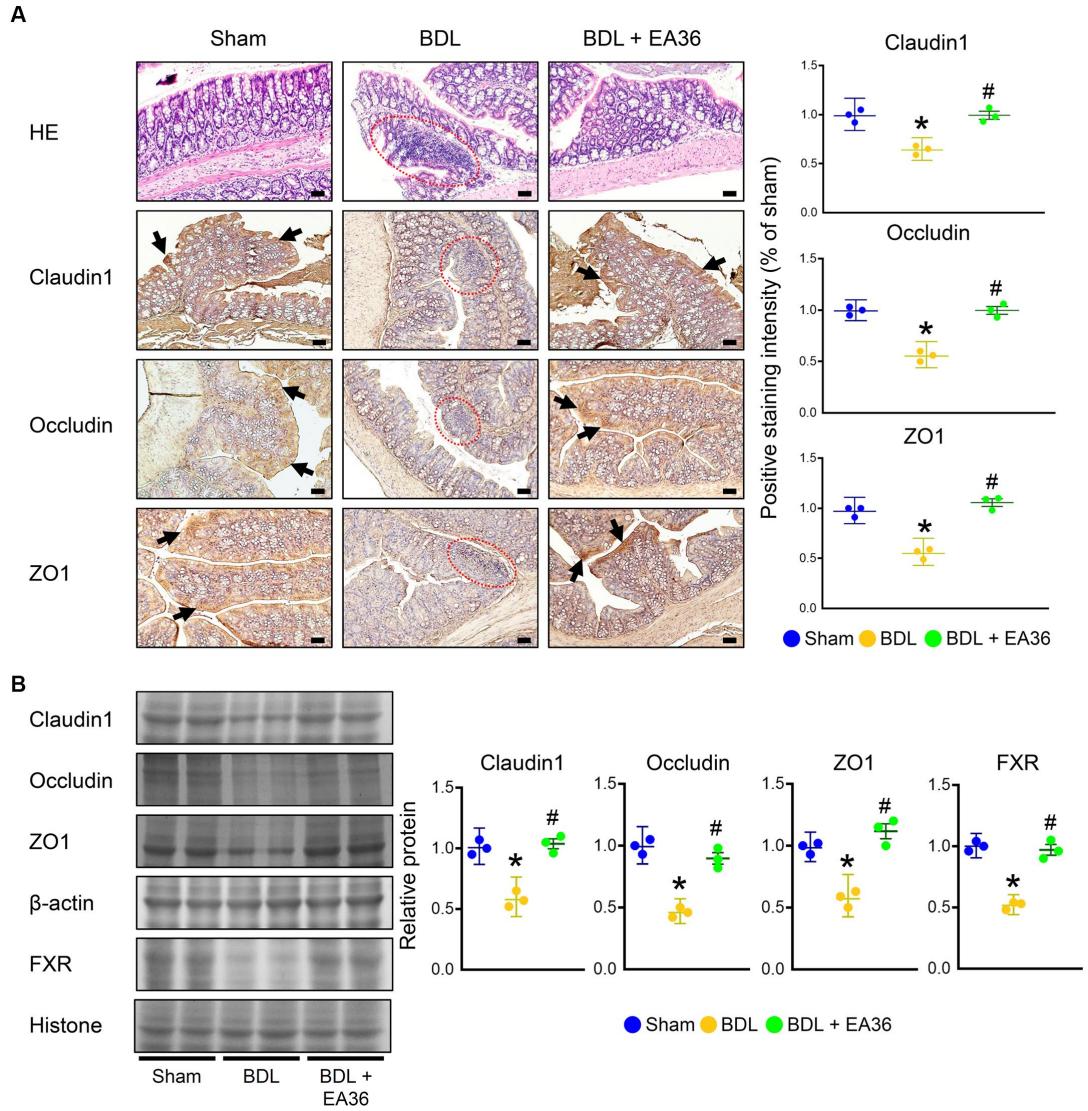
EA36 enhances tight junction in colon of BDL rats. **(A)** Colon tissues were sectioned and stained with hematoxylin-eosin (H&E), and immunostained with Claudin1, Occludin, and ZO1. The red circles denote colonic damage. Black arrows highlight positive staining. Scale bar: 50 μm. Right: Quantification of Claudin1, Occludin, and ZO1 protein levels by immunohistochemistry in graphs. **(B)** Quantification of Claudin1, Occludin, ZO1, and FXR protein levels by western blot of colon. Right graphs indicate quantification relative to β-actin and hiistone. The results represent mean ± SEM. ^*^p < 0.05, sham compared with BDL; ^#^*p* < 0.05, BDL compared with BDL + EA36.

The intestinal mucosa and colonic epithelium were both normal in the sham group. On the other hand, the colon of the BDL group showed severe histological damage as evidenced by epithelial atrophy, considerable inflammatory infiltration in the lamina propria, as well as erosion in the superficial epithelium. When compared to the sham group, these histological alterations were statistically significant. Notably, the treatment of EA36 reduced the histology score, as evidenced by better preserved epithelial morphology (Figure 4A). We further demonstrated that the barrier function was decreased in the rat colons due to BDL. Claudin1, occludin, and ZO1 levels were all significantly lower in the BDL-alone rats. Compared with the BDL-alone group, the levels of claudin1, occludin, ZO1, and FXR were significantly increased in the EA36 treatment groups (Figures 4A,B). We suggest that the effects of EA36 on BDL-induced intestinal barrier damage are connected to the control of the colonic mucosal barrier function, whereas the tight junction proteins levels of claudin1, occludin, ZO1, and FXR are critical in preserving the intestinal mucosal barrier function.

### 3.5. EA36 altered gut microbial composition in BDL rats

**Figure 5:**
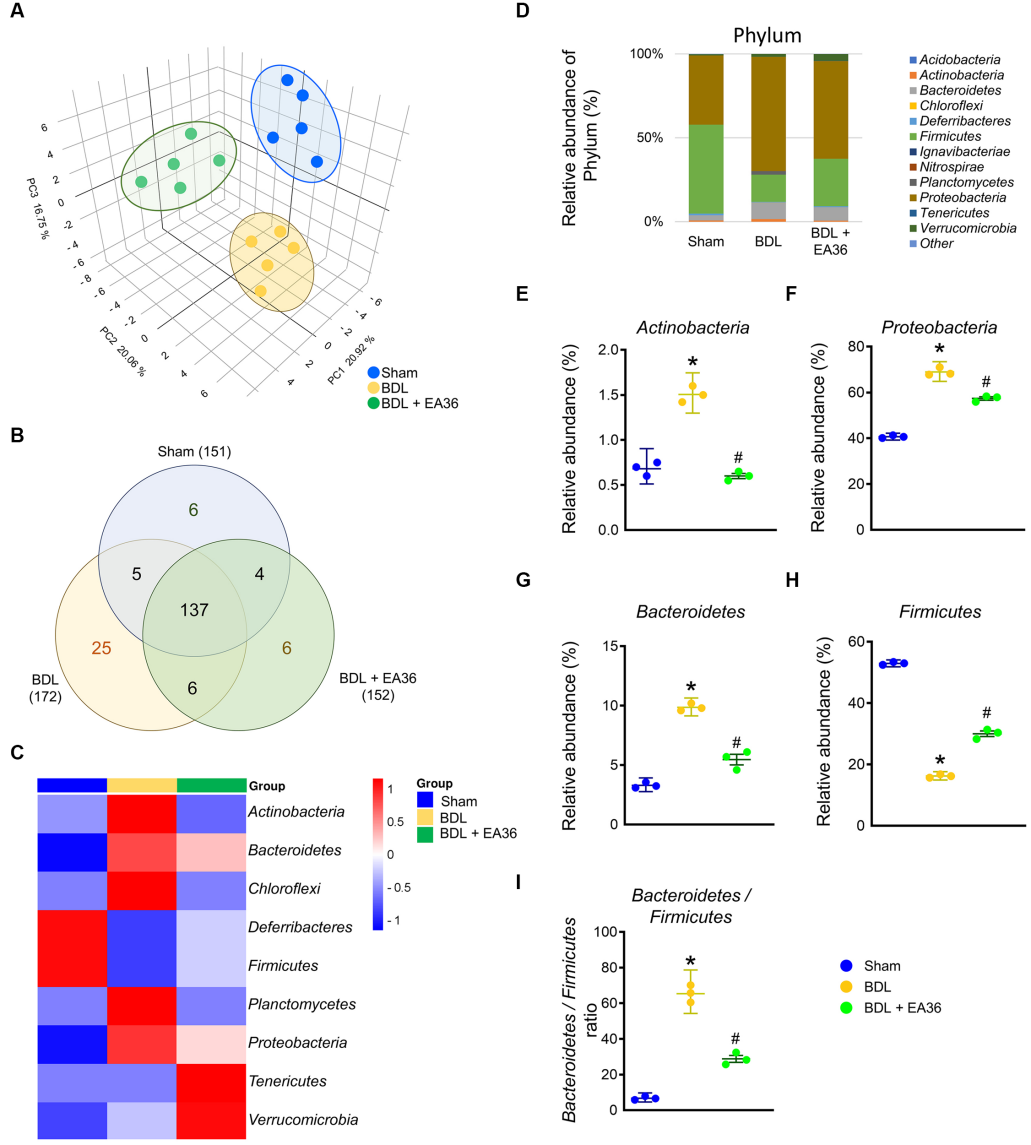
Transcriptomic analysis of fecal samples from sham, BDL, and BDL + EA36 rats. **(A)** 3D PCA plot based on bacterial sequence abundance in fecal content. Axes correspond to principal components 1 (*x*-axis), 2 (*y*-axis), and 3 (*z*-axis). **(B)** Venn diagram showing the total number of DEGs in sham, BDL and BDL + EA36 groups. The figure also demonstrates the distribution of operational taxonomic units (OTUs) among three groups is represented via the Venn diagram. **(C)** Heat map showing an overview of differentially expressed group sets with their associated microbiota in sham, BDL, and BDL + EA36 groups at the phylum level. **(D)** Phylum level microbiome composition in fecal. **(E–I)** Relative abundance of *Actinobacteria*, *Proteobacteria*, *Bacteroidetes*, *Firmicutes*, and *Bacteroidetes*/*Firmicutes* ratio. The results represent mean ± SEM. ^*^p < 0.05, sham compared with BDL; ^#^*p* < 0.05, BDL compared with BDL + EA36.

To determine the mechanism involved in EA36-mediated protection against BDL-induced colon injury, we assessed the effects of EA36 on microbiota composition in the colon by amplifying and analyzing amplicons from the V3-V4 region of the 16S rDNA gene. 3D Principal coordinates analysis showed that microbial communities in BDL rats clustered separately from sham group, and EA36 treatment attenuated the distinction (Figure 5A). A Venn diagram showed 151 features in the controls, 172 features in BDL rats, and 152 features in BDL rats treated with EA36 (Figure 5B). At the phylum level, variations in the microbial community composition were detected, sorted and heatmap by relative abundance in the samples (Figures 5C,D).

**Figure 6:**
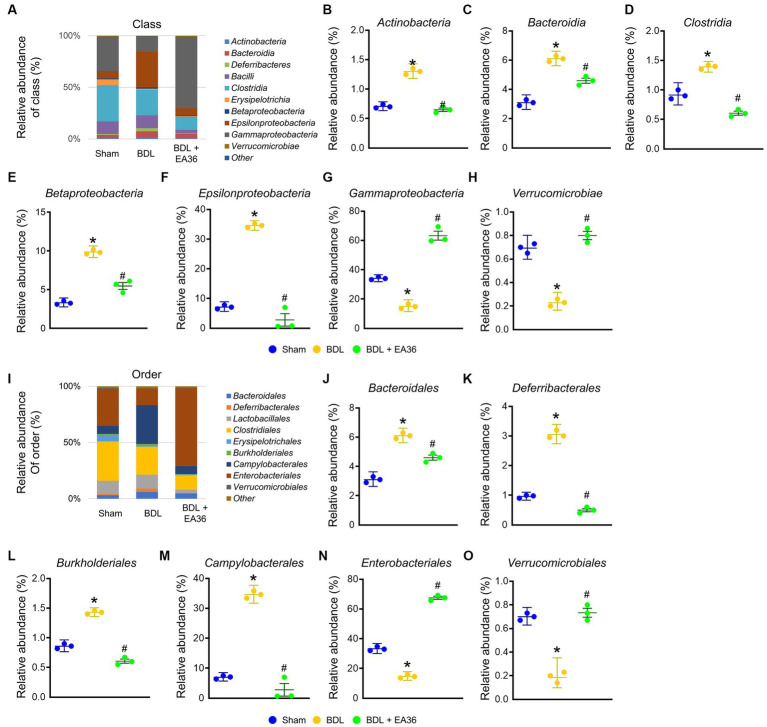
EA36 alters the class and order-level microbial composition in BDL rats. **(A)** Class level microbiome composition in feces. **(B–H)** Relative abundance of *Actinobacteria*, *Bacteroidia*, *Clostridia*, *Betaproteobacteria*, *Epsilonproteobacteria*, *Gammaproteobacteria*, and *Verrucomicrobiae*. **(I)** Microbiome composition at the order level in feces. **(J–O)** Relative abundance of *Bacteroidales*, *Deferribacterales*, *Burkholderiales*, *Campylobacterales*, *Enterobacteriales*, and *Verrucomicrobiales*. The results represent mean ± SEM. ^*^p < 0.05, sham compared with BDL; ^#^*p* < 0.05, BDL compared with BDL + EA36.

**Figure 7:**
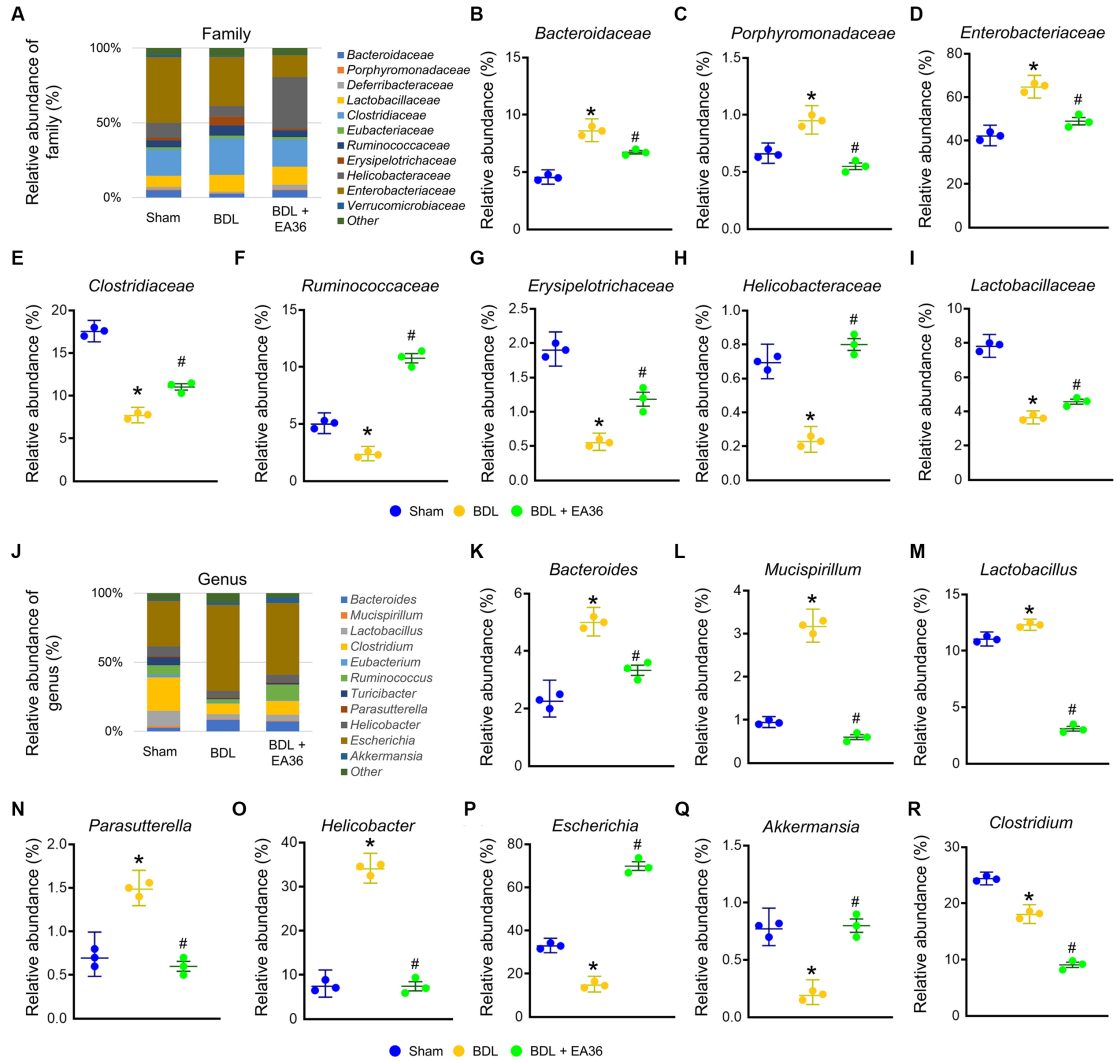
EA36 alters the family and genus-level microbial composition in BDL rats. **(A)** Family level microbiome composition in feces. **(B–I)** Relative abundance of *Bacteroidaceae*, *Porphyromonadaceae*, *Enterobacteriaceae*, *Clostridiaceae*, *Ruminococcaceae*, *Erysipelotrichaceae*, *Helicobacteraceae*, and *Lactobacillaceae*. **(J)** Genus level microbiome composition in feces. **(K–R)** Relative abundance of *Bacteroides*, *Mucispirillum*, *Lactobacillus*, *Parasutterella*, *Helicobacter*, *Escherichia*, *Akkermansia*, and *Clostridium*. The results represent mean ± SEM. ^*^p < 0.05, sham compared with BDL; ^#^*p* < 0.05, BDL compared with BDL + EA36.

**Figure 8:**
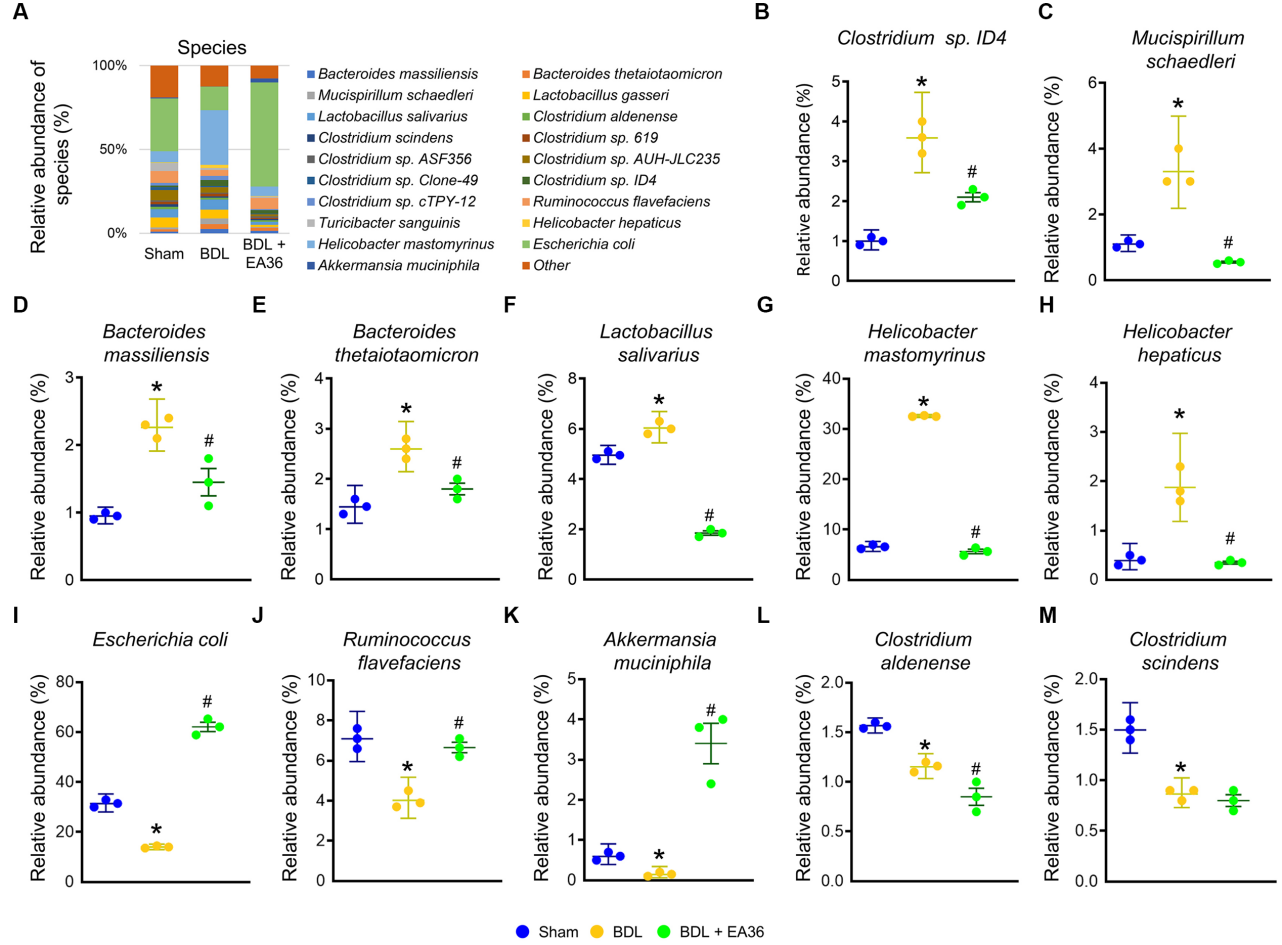
EA36 alters the species-level microbial composition in BDL rats. **(A)** Species level microbiome composition in feces. **(B–M)** Relative abundance of *Clostridium* sp. *ID4*, *Mucispirillum schaedleri*, *Bacteroides massiliensis*, *Bacteroides thetaiotaomicron*, *Lactobacillus salivarius*, *Helicobacter mastomyrinus*, *Helicobacter hepaticus*, *Escherichia coli*, *Ruminococcus flavefaciens*, *Akkermansia muciniphila*, *Clostridium aldenense*, and *Clostridium scindens*. The results represent mean ± SEM. ^*^p < 0.05, sham compared with BDL; ^#^*p* < 0.05, BDL compared with BDL + EA36.

**Figure 9:**
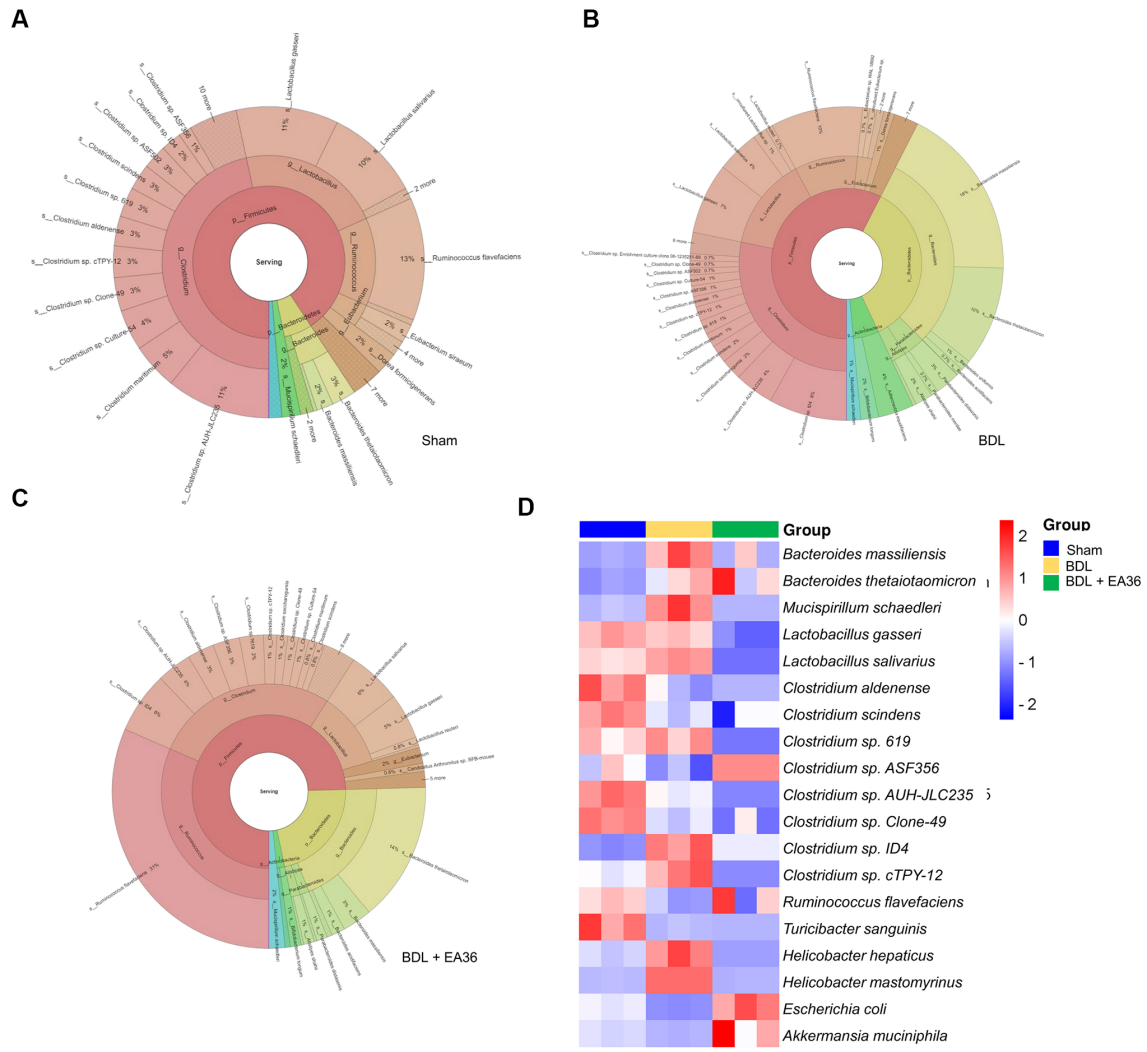
Interactive html result and heatmap representing the species-level microbial composition. **(A–C)** In the interactive html result, circles show different classification levels for the phylum, genus, and species (inside to outside) in the community. **(D)** Heat map showing an overview of differentially expressed group sets with their associated microbiota in sham, BDL and BDL + EA36 groups from the species level.

According to the species annotation, the statistical number of sequences of every sample at each classification level (phylum, class, genus, and species) was calculated. We created a bar plot to present the results. All sequences were classified and identified from phylum to species level (Figures 4–9). The 16S rRNA profiles of each experimental group in the colon were very dissimilar, even in phylum-level distributions (Figure 5D). BDL induced Actinobacteria, Proteobacteria, Bacteroidetes, Bacteroidetes/Firmicutes ratio, and reduced Firmicutes relative abundance (Figures 5E–I), and EA36 treatment reversed the gut microbiota abundance.

### 3.6. EA36 altered gut microbial composition at class and order levels

At class level, BDL induced Actinobacteria, Bacteroidia, Clostridia, Betaproteobacteria, Epsilonproteobacteria, and reduced Gammaproteobacteria, Verrucomicrobiae relative abundance (Figures 6A–H). At order level, BDL induced Bacteroidales, Deferribacterales, Burkholderiales, Campylobacterales, and reduced Enterobacteriales, Verrucomicrobiales relative abundance (Figures 6I–O). The effects were improved following treatment with the EA36 at class and order levels.

### 3.7. EA36 altered gut microbial composition at family and genus levels

At family level, EA36 treatment reversed BDL-increased the relative abundances of Bacteroidaceae, Porphyromonadaceae, and Enterobacteriaceae and decreased the relative abundances of Clostridiaceae, Ruminococcaceae, Erysipelotrichaceae, Helicobacteraceae, and Lactobacillaceae (Figures 7A–I), and the relative abundances of the gut microbial were reverse following EA36 treatment. At genus level, BDL-induced *Bacteroides*, *Mucispirillum*, *Lactobacillus*, *Parasutterella*, *Helicobacter*, and reduced *Escherichia*, *Akkermansia*, and *Clostridium* relative abundance (Figures 7J–R). The effects were improved following treatment with the EA36 at family and genus levels.

### 3.8. EA36 altered gut microbial composition at species level

Furthermore, at the phylum, class, order, family, genus, and species levels, the bacterial composition varied in the BDL treatment group compared to the sham group. EA36 treatment reversed BDL-increased Clostridium sp. ID4, *Mucispirillum schaedleri*, *Bacteroides massiliensis*, *Bacteroides thetaiotaomicron*, *Lactobacillus salivarius*, *Helicobacter mastomyrinus*, *Helicobacter hepaticus*, and reduced *Escherichia coli*, *Ruminococcus flavefaciens*, *Akkermansia muciniphila*, *Clostridium Aldenense*, and *Clostridium scindens* relative abundance (Figures 8A–M), with the exception of the relative abundance of *Clostridium aldenense* and *Clostridium scindens*.

### 3.9. EA36 altered html result and heatmap in the species-level microbial composition

We then investigated at the taxonomy of the gut microbiota as a whole. The circles in the interactive HTML result represent the various categorization levels of the community, from the phylum, genus, and species (inside to outside), and the dimension of the sector represents the proportion of various OTU annotation results (Figures 9A–C). Comparative to the BDL group, the EA36 treatment effectively reversed the dysbiosis profile (Figures 9A–C). In BDL rats, EA36 significantly altered the content of the gut microbiota, and the proportion of the microbiota returned to their original state. At the species level, variations in the microbial community composition were detected, heatmap by relative abundance in the samples (Figure 9D).

## 4. Discussion

The major findings of this study relate to a potential mechanism of EA that links gut microbiota and angiogenesis signaling related vascular hyporeactivites in cholestatic liver injury with PHT. The interpretations are discussed in the following paragraphs.

Bile duct ligation is a surgical procedure that tying off of the bile ducts to prevent the flow of bile from the liver. This procedure can cause a number of complications, including hepatic injury. The damage caused by BDL can include inflammation, fibrosis, and cirrhosis (Chang et al., 2019; Chen et al., 2022). This procedure can be used to induce cholestasis, a condition in which the flow of bile is impaired, resulting in accumulation of bile acids and other components in the liver, leading to liver damage. Induction of cholestasis by BDL has been used in several studies to induce liver injury in animal models to study acute and chronic hepatic injury (Chang et al., 2019; Chen et al., 2022). When cholestasis occurs, the bile acids are unable to flow into the intestines, resulting in the disruption of intestine microbiota caused by cholestasis can lead to various complications such as bacterial overgrowth, increased permeability of the intestine, and changes in the composition of the gut microbiota. These changes can further contribute to liver injury and lead to the buildup of toxins in the bloodstream, which can lead to further complications.

The gut microbiota plays a vital role in food digestion, metabolism, immune regulation, and even neurocognitive performance. The above physiological processes show that the gut microbiota has diurnal characteristics (Thaiss et al., 2014). In addition, intestinal epithelial cells rhythmically sense microbial changes, which are very important for the homeostasis of the intestinal epithelium (Mukherji et al., 2013). In the current study, the genes level of *Bacteroidetes*, *Betaproteobacteria*, *Epsilonproteobacteria*, *Bacteroides thetaiotaomicron*, *Lactobacillus salivarius*, and *Helicobacter hepaticus* were significantly elevated due to BDL, while EA36 treatment markedly reduced these microbiome genes levels. EA36 modulates the gut microbiota composition and increases the abundance of beneficial bacteria such as *Bacteroidetes*, *Betaproteobacteria*, *Epsilonproteobacteria*, and *Lactobacillus salivarius*, which have anti-inflammatory properties (Zafar and Saier, 2021). EA36 also increases the expression of tight junction proteins that regulate the permeability of the gut and reduce inflammation. Decreased the above microbiome genes levels due to cholestasis, which tended to support the hypothesis that EA36 ameliorates cholestatic liver injury through modulation of microbiota abundance or diversity and its anti-inflammatory effect. *Bacteroidetes* can produce anti-inflammatory molecules that can reduce inflammation by directly blocking the production of pro-inflammatory cytokines (Stojanov et al., 2020). *Bacteroidetes* also produce metabolites that can help maintain gut barrier integrity, which can reduce inflammation (Khan et al., 2020; Ghosh et al., 2021). Nevertheless, most studies have demonstrated that *Bacteroidetes* bacteria exhibit pro-inflammatory properties due to endotoxins and influence cytokine production, contributing to IBD (Stojanov et al., 2020). Furthermore, *Firmicutes* bacteria exhibit anti-inflammatory effects and can alleviate the progression of IBD. The *Firmicutes*/*Bacteroidetes* (F/B) ratio is widely accepted to have an important influence in maintaining normal intestinal homeostasis (Stojanov et al., 2020). EA36 balancing the intestinal ecosystem is an important aspect of maintaining intestine barrier function, and may possess the therapeutic strategy to achieve an appropriate F/B ratio. The most variable phylum was demonstrated to be *Proteobacteria*, which contributes to dysbiosis (Shin et al., 2015) and is correlated with a decrease in *Firmicutes* and general microbial diversity in inflammatory bowel disease (IBD; Morgan et al., 2012).

In accordance with previous reports, our results showed that the raised levels of *Bacteroidetes* in the fecal are attributable to diminish cholestasis. The treatment of EA36 significantly attenuated the *Bacteroidetes* levels. The attenuation of microbiome levels byEA36 treatment could partially be related to its anti-inflammation and remodel microbiota ecology. Another explanation for this statistically significant decrease in the above microbiota genes seen in the EA36 treated rats compared with the BDL rats is the effect of EA36 on the gut tight junction proteins, whereby EA36 may maintain the function of intestine barrier permeability during cholestasis.

Microbiota can be prescribed for PHT. Microbiota have been shown to improve gut health, which can help reduce portal pressure (Baffy, 2019). Microbiota have anti-inflammatory properties, which can help reduce inflammation associated with PHT (Baffy, 2019; Gedgaudas et al., 2022). Microbiota can help increase nutrient absorption and reduce the risk of liver damage associated with portal hypertension (Simbrunner et al., 2019). Microbiota can help protect the gut lining, reducing the risk of gastrointestinal bleeding associated with PHT (Simbrunner et al., 2019). Microbiota can help reduce the risk of bacterial overgrowth and infection, which can contribute to the development of PHT (Gedgaudas et al., 2022). Indeed, our results demonstrated that both of the portal pressure and SMA blood flow values in BDL rats were reversed by EA36 are associated with gut microbiota. Studies have shown that acupuncture can help reduce PHT in patients by reducing their blood pressure, improving blood circulation, and reducing inflammation (Yang et al., 2018; Fan et al., 2020). It can also help reduce the symptoms of PHT, such as nausea, vomiting, and abdominal pain (Ouyang and Chen, 2004). EA36 for PHT may involve modulating the microbiota by stimulating certain acupoints ST36 (Chang et al., 2019; Chen et al., 2022). EA36 may help to reduce PHT by modulating the composition of the gut microbiota. Our study has shown that EA36 can increase the diversity and abundance of beneficial bacterial species, while decreasing the abundance of potentially harmful ones (Liu et al., 2018). This can help to reduce inflammation and improve the overall health of the gut microbiome, which can in turn reduce PHT in current results. In addition, EA for PHT may modulate the gut microbiota, which in turn attenuates vascular activity. This can help to reduce the pressure in the portal vein, helping to reduce symptoms associated with PHT. Additionally, EA36 may help to reduce inflammation, improve blood flow, and reduce cholestatic stress, all of which can help to reduce the symptoms of portal hypertension. Additionally, EA36 enhances tight junction in colon of BDL rats. EA36 has been studied for its potential to attenuate intestinal permeability in animal models of colitis (Liu et al., 2020, 2022). Our current studies have shown that EA36 treatment can reduce intestinal barrier dysfunction and improve intestinal permeability in rats with PHT. EA36 treatment make to reduce the expression of inflammatory cytokines levels, and reduce vascular endothelial growth factor (VEGF) expression and upregulate the expression of tight junction proteins, thereby improving the integrity of the intestinal epithelial barrier.

Portal hypertension may modulate the gut microbiota, which could result in vascular hyporeactivity (Baffy, 2019). This is because the alteration of the gut microbiota could lead to an increase in pro-inflammatory cytokines and endotoxins, which can affect vascular reactivity and cause fail of vasoconstriction (Simbrunner et al., 2019; Mitten and Baffy, 2022). These changes in the gut microbiota could potentially lead to increased levels of PHT, which could then further contribute to vascular hyporeactivity. On the other hand, PHT may modulate the intestine vagus nerve, which in turn can cause vascular hyporeactivity (Bockx et al., 2012; Zong et al., 2014). This can also lead to an impaired ability to regulate the blood vessels, resulting in increased systemic vascular resistance and increased blood pressure in the portal venous system. Vagus nerve stimulation has been shown to reduce vascular hyporeactivity by inducing vasodilation and decreasing vascular resistance (Dümcke and Møller, 2008). This is largely due to the release of nitric oxide from the nerve endings of the vagus nerve, which acts as a vasodilator (Li and Olshansky, 2011; Tse, 2017). Therefore, stimulation of the intestine vagus nerve can play a role in improving vascular hyporeactivity. Studies have suggested that stimulating the vagus nerve may help to reduce PHT, a condition where the pressure in the portal vein, which carries blood from the digestive organs to the liver, is higher than normal (Bockx et al., 2012). EA36 may help to regulate digestion, heart rate, and blood pressure. The EA36 stimulation plays a major role in the communication between the gut microbiome and the vascular activities. It is responsible for sending signals from the gut to the vessels. This communication is essential for maintaining a healthy gut microbiome, as it helps regulate the balance of beneficial and pathogenic microbes in the gut. Acupuncture has been shown to stimulate the vagus nerve (Liu et al., 2021), and by doing so, it can help to maintain overall health and potentially attenuate PHT. By stimulating the vagus nerve, EA36 may possibly increase parasympathetic activity, decrease sympathetic activity, and promote the release of neurotransmitters and hormones that can reduce inflammation and improve the health of the gastrointestinal tract. In addition, acupuncture can help to improve blood circulation and reduce the risk of spasms and blood clots, which can help reduce the risk of PHT.

Vascular angiogenesis refers to the growth of new blood vessels, usually from existing ones. It is important in the pathophysiology of PHT because it is a major factor in the development of portosystemic shunts, which are abnormal connections between the portal and systemic veins (Iwakiri and Trebicka, 2021). These abnormal connections allow blood to bypass the normal flow through the liver, leading to increased pressure in the portal vein. In some cases, an increase in vascular angiogenesis can contribute to the development of PHT. This is because new blood vessels can form in response to increased pressure in the portal vein, leading to an increase in blood flow and further increasing the pressure. In PHT, the increased pressure in the portal vein results in increased resistance to blood flow, causing increased levels of angiogenesis (Iwakiri and Trebicka, 2021; Königshofer et al., 2022). This increased angiogenesis leads to the formation of new, smaller vessels, which are less able to withstand the increased pressure. This, in turn, leads to a decrease in the amount of blood flow to the liver and other organs, resulting in vascular hyporeactivity. Vascular hyporeactivity is a condition characterized by decreased vascular responsiveness to vasoactive agents, such as vasoconstrictors and vasodilators. In PHT animal models, vascular hyporeactivity is observed as a result of increased wall shear stress and decreased nitric oxide production in the liver (Langer and Shah, 2006; Königshofer et al., 2022). This leads to a decrease in vascular tone and a reduction in vasomotor responsiveness, resulting in excessive vasodilation and blood pooling in the congested hepatic circulation. Here, we found that the ability of EA36 to prevent BDL-induced vascular angiogenesis is consistent with its inhibited role in the diminution of ICAM1, VCAM1, TGFβR2, and VEGFR1 in the vasculature of BDL rats, specifically its ability to suppress the proinflammatory cytokines were significantly when compared with BDL rats. This action is believed to be dependent not only on its modulation effect on microbiota abondance, but also on its inhibition of angiogenesis pathways.

The microbiome is an incredibly complex and delicate system that make it is easily disrupted. In current study, the potential anti-inflammatory effect of EA36 may through vagus nerve stimulation, which is able to dampen peripheral inflammation and to decrease intestinal permeability, thus very probably modulating microbiota composition. It is a notable worthy that several reports showed that the vagus nerve is able to sense the microbiota, to transfer this gut information to the central nervous system where it is integrated, and then to generate an adapted or inappropriate response; the latter could perpetuate a pathological condition of the digestive tract or favor neurodegenerative disorders (Eisenstein, 2016; Tse, 2017). On the other hand, stress inhibits the vagus nerve and has deleterious effects on the gastrointestinal tract and on the microbiota, and is involved in the pathophysiology of gastrointestinal disorders such as irritable bowel syndrome (IBS) and inflammatory bowel disease (IBD) which are both characterized by a dysbiosis (Bonaz et al., 2018). Although the vagus nerve plays a critical role in regulating the body’s microbiome but using a vagus nerve inhibitor in our study would likely have a significant and unpredictable effect on the microbiome. The vagus nerve inhibitors can have a direct impact on the microbiome, making it difficult to accurately assess the effects of the EA36 on the microbiome. Furthermore, using a vagus nerve inhibitor may disrupt the delicate balance of the microbiome, causing unwanted side effects that could interfere with the EA36 study results. For above reasons, we do not use a vagus nerve inhibitor to block EA36 action in our study.

Therefore, the beneficial effects of EA36 in PHT may be mediated, at least in part, through the modulation of intestine microbiota and vascular hyporeactivity with subsequent amelioration of the hyperdynamic circulation. In conclusion, this study illustrates the positive role of EA in a cholestatic model of PHT, and shown potential inhibition of intestine inflammation, significantly blunts the endotoxemia-mediated intestine permeability disruption and vessels contraction via a mechanism that involves angiogenesis-dependent activation of proinflammatory cytokines in these PHT animals. We conclude that EA may possess therapeutic properties that can mitigate the development of the hemodynamic change that characterize PHT animals. This molecular action suggests that AP may prove useful as a research tool or in clinical applications.

## Data availability statement

The datasets presented in this study can be found in online repositories. The names of the repository/repositories and accession number (s) can be found at: Sequence Read Archive (SRA; https://www.ncbi.nlm.nih.gov/sra/PRJNA957945) and BioSample (accession nos. SAMN34277024, SAMN34277025, and SAMN34277026).

## Ethics statement

Animal studies were approved by the Animal Experiment Committee of Chang Gung University (IACUC approval no. CGU15-088).

## Author contributions

P-YH, H-ML, and T-YL conceived and designed the research and wrote the first draft of the manuscript. P-YH and H-ML conducted experiments. Y-RK assisted in the establishment of a model and the improvement of strategies. Z-YC and P-YH performed statistical analyses and wrote the sections of the manuscript. All authors contributed to the article and approved the submitted version.

## Funding

This research was funded by Nation Science and Technology Council, Taipei, Taiwan (funding numbers: NSTC 102-2320-B182-015-MY3, 103-2320-B182-002-MY3, 106-2320-B-182-005-MY3, and 109-2320-B-182-023-MY3), Chang Gung Memorial Hospital, Linkou, Taiwan (funding numbers: CMRPD1B0261, CMRPD1B0262, CMRPD1D0351, and CMRPD1D0352), and Chang Gung Memorial Hospital, Keelung, Taiwan (funding number: CMRPG2M0351).

## Conflict of interest

The authors declare that the research was conducted in the absence of any commercial or financial relationships that could be construed as a potential conflict of interest.

The reviewer G-HL declared a shared affiliation with the authors T-YL, P-YH, and H-ML to the handling editor at the time of review.

## Publisher’s note

All claims expressed in this article are solely those of the authors and do not necessarily represent those of their affiliated organizations, or those of the publisher, the editors and the reviewers. Any product that may be evaluated in this article, or claim that may be made by its manufacturer, is not guaranteed or endorsed by the publisher.
